# Evaluation of cross-platform compatibility of a DNA methylation-based glucocorticoid response biomarker

**DOI:** 10.1186/s13148-022-01352-1

**Published:** 2022-10-28

**Authors:** Emily Tang, John K. Wiencke, Gayathri Warrier, Helen Hansen, Lucie McCoy, Terri Rice, Paige M. Bracci, Margaret Wrensch, Jennie W. Taylor, Jennifer L. Clarke, Devin C. Koestler, Lucas A. Salas, Brock C. Christensen, Karl T. Kelsey, Annette M. Molinaro

**Affiliations:** 1 Department of Epidemiology and Biostatistics, University of California San Francisco, San Francisco, CA USA; 2 Department of Neurological Surgery, University of California San Francisco, San Francisco, CA USA; 3 Department of Neurology, University of California San Francisco, San Francisco, CA USA; 4 Department of Biostatistics and Data Science, University of Kansas Medical Center, Kansas City, KS USA; 5 Department of Epidemiology, Geisel School of Medicine, Dartmouth College, Lebanon, NH USA; 6 Departments of Molecular and Systems Biology, and Community and Family Medicine, Geisel School of Medicine, Dartmouth College, Lebanon, NH USA; 7 Departments of Epidemiology and Pathology and Laboratory Medicine, Brown University, Providence, RI USA

**Keywords:** DNA methylation, Whole blood, Cord blood, Dexamethasone, Glucocorticoid, Algorithmic biomarker, 450K versus 850K

## Abstract

**Background:**

Identifying blood-based DNA methylation patterns is a minimally invasive way to detect biomarkers in predicting age, characteristics of certain diseases and conditions, as well as responses to immunotherapies. As microarray platforms continue to evolve and increase the scope of CpGs measured, new discoveries based on the most recent platform version and how they compare to available data from the previous versions of the platform are unknown. The neutrophil dexamethasone methylation index (NDMI 850) is a blood-based DNA methylation biomarker built on the Illumina MethylationEPIC (850K) array that measures epigenetic responses to dexamethasone (DEX), a synthetic glucocorticoid often administered for inflammation. Here, we compare the NDMI 850 to one we built using data from the Illumina Methylation 450K (NDMI 450).

**Results:**

The NDMI 450 consisted of 22 loci, 15 of which were present on the NDMI 850. In adult whole blood samples, the linear composite scores from NDMI 450 and NDMI 850 were highly correlated and had equivalent predictive accuracy for detecting DEX exposure among adult glioma patients and non-glioma adult controls. However, the NDMI 450 scores of newborn cord blood were significantly lower than NDMI 850 in samples measured with both assays.

**Conclusions:**

We developed an algorithm that reproduces the DNA methylation glucocorticoid response score using 450K data, increasing the accessibility for researchers to assess this biomarker in archived or publicly available datasets that use the 450K version of the Illumina BeadChip array. However, the NDMI850 and NDMI450 do not give similar results in cord blood, and due to data availability limitations, results from sample types of newborn cord blood should be interpreted with care.

**Supplementary Information:**

The online version contains supplementary material available at 10.1186/s13148-022-01352-1.

## Background

DNA methylation is a stable epigenetic mark in humans that can identify risk factors, genetic differences, and has been shown to associate with survival outcomes. Regions of the DNA where methylation can act coordinately with other chromatin factors to alter gene regulation are primarily CpG sites, where a cytosine and guanine are base paired [1]. Across different sample types or different individuals, differentially methylated regions of one or more CpG sites can potentially lead to methylation biomarkers that may be useful in predicting events such as disease progression and responses to therapies [2]. A broadly applied platform for genome-wide DNA methylation analysis is the Illumina Infinium BeadChip technology [3]. The Illumina HumanMethylationEPIC array (i.e., 850K array; introduced in 2015) measures over 850,000 CpG sites in the human genome and builds on the Illumina HumanMethylation450 array (450K array), which measured 485,512 CpG sites. As the technology improves and increases the coverage of CpGs that it can measure, findings and biomarkers developed from the most recent 850K microarray version may have limited backward compatibility with datasets and samples run on its previous versions. As of May 2022, 82% of publicly available microarray data on the EWAS Data Hub are from the 450K array, resulting in a plethora of archived studies and samples, so it is important to validate biomarkers built on the newer arrays on the older version arrays [4].

Glucocorticoids are commonly used to treat inflammation for a myriad of diseases; in glioma patients, synthetic glucocorticoids such as dexamethasone (DEX) are used to treat swelling of the brain [5]. Despite their benefits, exogenous glucocorticoids like DEX give rise to highly variable responses among people, and patients may experience adverse events. In pregnancy, glucocorticoids play an important role in fetal programming, and cortisol plays a key role in antenatal development [6]. Antenatal glucocorticoid treatment is routinely given to mothers at risk for preterm birth, but this suppresses the hypothalamic–pituitary–adrenal (HPA) axis, which can lead to adrenal insufficiency, as the HPA axis is a major physiological stress response system that is important in maintaining homeostasis. Furthermore, exposure to stress during intrauterine life and other factors that raise maternal cortisol levels can result in excess cortisol exposure to the fetus. Toward the end of pregnancy, fetal exposure to maternal cortisol increases as the levels of the placental enzyme decrease. Thus, fetal exposure to elevated maternal cortisol during the third trimester is associated with alterations in infant feedback inhibition, and in the HPA axis [7]. Several studies have observed adrenal insufficiencies, neurodevelopmental delays, and other long-term consequences of elevated prenatal exposures from endogenous maternal cortisol levels [6, 8–10].

Studies suggest that there is epigenetic regulation of the glucocorticoid receptor [11–13]. In one study, researchers identified a methylation predictor of glucocorticoid excess and found that the *FKBP5* gene locus had a concentration of hypomethylated CpG sites among those with Cushing’s syndrome. *FKBP5* is well documented in regulating glucocorticoid receptor sensitivity, and it is also a component in the neutrophil dexamethasone methylation index (NDMI), a pharmacodynamic DNA methylation biomarker that characterizes an individual patient’s exposure and response to glucocorticoids [13]. The NDMI accurately predicts exposure to DEX at the time of blood draw in glioma patients and non-glioma controls [14]. However, this biomarker was built using the Illumina HumanMethylationEPIC array and cannot be used on data from earlier generations of the Illumina methylation arrays (e.g., 450K array). Given the potential clinical and biological applications of the NDMI coupled with the wealth of publicly available 450K data, we sought to reconstruct the NDMI biomarker using CpG loci on the 450K array (NDMI 450).

The NDMI could yield potential insight into the pathophysiology of adrenal disorders and other factors involved in the glucocorticoid pathway, such as those involved in reproductive outcomes. Within the first few days after birth, a blood test is run to hormone-related, rare genetic, and metabolic conditions that could potentially cause serious health problems to ensure a timely diagnosis [15]. Genetic variations of neonatal *FKBP5* have been associated with mental health outcomes and fetal hippocampal development with increased antenatal maternal depressive symptoms [16]. Exposure to betamethasone, a synthetic glucocorticoid, was also found to be significantly associated with lower placenta methylation at the enhancer site of *FKBP5* and consequently higher expression of *FKBP5* in placenta samples [17]. Using a blood-based biomarker capable of capturing inter-individual variation in glucocorticoid exposure and response could help clarify the complex relationship between maternal risk factors and developmental health outcomes and identify adrenal disorders beyond the standard blood test.

Immune responses also are linked to glucocorticoids and play an important role during pregnancy. Further, metabolic disturbances such as gestational diabetes, anxiety, and preterm birth can lead to neonatal complications and problems with development. In newborns born to individuals with gestational diabetes mellitus (GDM), γδ T cell and NK cell levels were found to be higher, and CD4T cell proportions lower than that of healthy pregnant women [18]. Immune cells in term and preterm labor participate in various phases of the pregnancy. Compared to term infants, preterm infants were observed to have lower proportions of CD56^bright^ NK cells, CD8+ T cells, and γδ T cells, and increased levels of CD4+ T cells and transitional B cells [19].

Here, we created a DEX response biomarker using CpG probes contained on the 450K array. The two aims of this study were to compare the predictive accuracy of discriminating clinical use of DEX with the new 450K biomarker compared to that of the 850K biomarker and to evaluate the performance of the 450K biomarker with pregnancy variables where 850K data were not available.

## Results

**Fig. 1:**
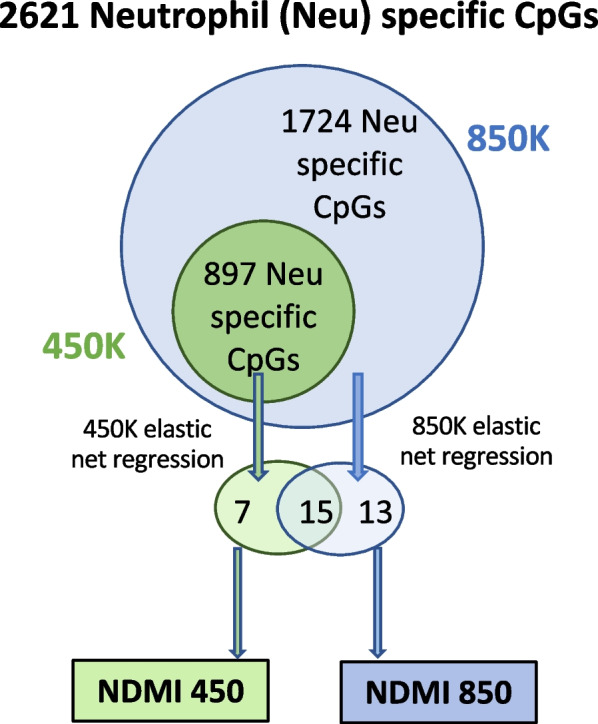
Workflow. A Venn diagram demonstrating the neutrophil-specific CpG probes present on 450K and 850K platforms, the output of elastic net regression, and calculation of the linear composite score for NDMI 450 and NDMI 850. Of the 2621 neutrophil-specific probes of interest on the 850K array, 897 probes were available in the 450K. While for NDMI 850, elastic net chose 28 CpG probes as discriminative of DEX use, 22 were chosen for NDMI 450. 15 of these probes were shared between the two algorithms. The coefficients of the respective CpG probes were used to calculate the linear composite score (NDMI score) for each patient

*Elastic net regression modeling with neutrophil-specific CpG probes associated with DEX on the 450K array* Out of the 2621 neutrophil-specific CpG probes from the 850K array that had statistically significant interactions with the exposure to DEX (*p* < 0.01), 897 of the 2621 probes (34.2%) were available on the 450K array. In elastic net regression models using the 897 neutrophil-specific loci available on the 450K array to predict DEX at blood draw among the IPS pre-surgery samples, 22 CpGs with nonzero coefficients were chosen from this NDMI 450 model, 28 CpGs were selected by the NDMI 850 model, and 15 of these loci overlapped between the two models (Fig. 1, Additional file 2: Supp. Table S1). The 13 loci that are unique to NDMI 850 are also unique to the 850K array, and the 7 loci unique to NDMI 450 are present on the 850K array. The NDMI 450 is capable of classifying DEX use from a linear composite score, which we will term as the NDMI 450 score and can be calculated as follows:

$$p = {\text{probability}}\;{\text{of}}\;{\text{taking}}\;{\text{DEX}}\;{\text{at}}\;{\text{blood}}\;{\text{draw}} = \frac{{e^{x} }}{{1 + e^{x} }},{\text{where}}\;x = {\text{NDMI }}450$$

$${\text{NDMI }}450\;{\text{score}} = \beta_{0} + \left( {\beta_{1} * cg00052684} \right) + \left( {\beta_{2} * cg13077031} \right) + \cdots + \left( {\beta_{22} * cg27094376} \right)$$

For each sample, the linear composite score is calculated by taking the sum of the intercept ($\beta_{0}$) and the summation of the 22 beta coefficients for each CpG and their covariate values. Each beta coefficient represents the change in mean response to DEX, per unit increase in methylation for that particular CpG, when all other 21 CpGs are held constant.

**Fig. 2:**
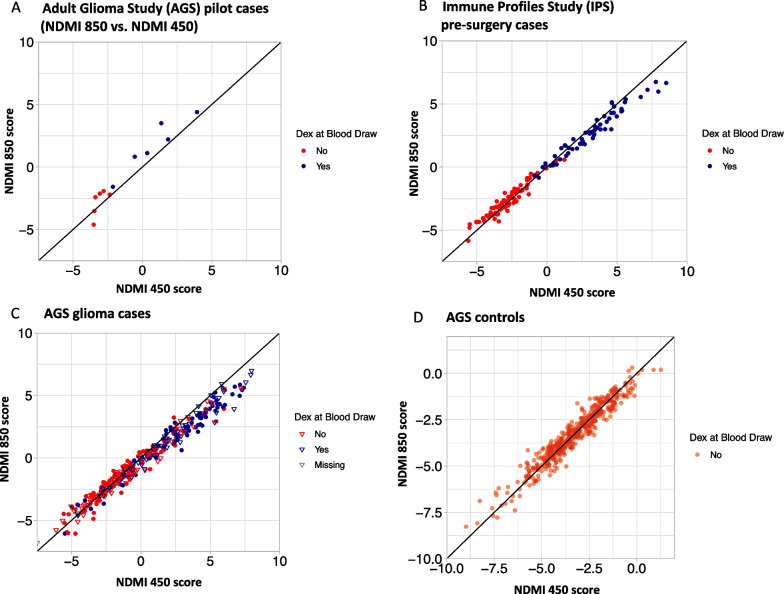
High correlation between NDMI 450 and NDMI 850 scores in the IPS and AGS samples. Those taking dexamethasone at the time of blood draw are in blue, and those who are not are in red. **A** In the AGS pilot cases comparing the same set of patients run on both arrays, the Pearson correlation was *r* = 0.97 (*p* < 0.0001). **B** In IPS training data, correlation was *r* = 0.99 (*p* < 0.0001). **C** In AGS glioma cases, those with IDH/1p19q/TERT-WT WHO 2016 classification of gliomas denoted by circles had *r* = 0.97 (*p* < 0.0001), and other AGS gliomas denoted by triangles had *r* = 0.98 (*p* < 0.0001). **D** In the adult controls, the correlation was *r* = 0.97 (*p* < 0.0001)

*Cross-platform compatibility of NDMI in adult whole blood samples* We first evaluated the NDMI in the 12 pilot samples from the AGS study based on only the 15 overlapping probes that are represented on the 450K array (reduced NDMI 850). We found that although the Pearson correlation to NDMI 850 scores was high (*r* = 0.99, *p* < 0.0001), the value of the reduced NDMI 850 scores was biased considerably using only the overlapping probes (Additional File 1: Supp. Fig. S1). For NDMI 850, the mean (standard deviation) of the scores was − 0.53 (2.86), and for the reduced NDMI 850, the mean was 4.10 (1.86), making the comparison of scores across the two platforms problematic. We then evaluated the NDMI 450 biomarker. The pilot cases that were run on both arrays had a correlation of *r* = 0.97 (*p* < 0.0001) between the NDMI 450 and NDMI 850 scores. Therefore, we identified additional CpG sites that retain the high correlation but improved the accuracy of the 450K based NDMI. In training and test datasets, the NDMI 450 and NDMI 850 scores were highly correlated. In the training data of IPS pre-surgery samples, correlation was *r* = 0.99 (*p* < 0.0001). In AGS glioma cases, those with IDH/1p19q/TERT-WT WHO 2016 classification of glioma had *r* = 0.97 (*p* < 0.0001), and other AGS glioma cases had *r* = 0.98 (*p* < 0.0001). In the adult controls, the correlation was 0.97 (*p* < 0.0001) (Fig. 2). The agreement between NDMI 850 and NDMI 450 scores was examined with Bland–Altman analysis (Additional file 1: Supp. Fig. S2).

**Fig. 3:**
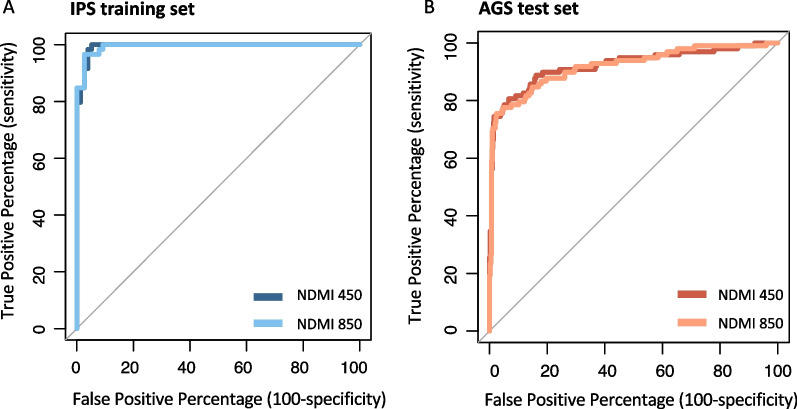
Comparable classification accuracy of DEX exposure by NDMI 450 in IPS and AGS patients. **A** In the training set (IPS pre-surgery samples), the AUROC of the NDMI 450 (blue) is compared to that of the NDMI 850 (the gold standard, light blue), which was 99.4% (95% CI 98.7%, 100%). **B** In the test set of 552 AGS cases and controls used to evaluate NDMI 850, the AUROC was 92.3% (95% CI 88.5%, 96%) for the NDMI 450 (red) and 91.9% (95% CI 88.3%, 95.6%) for the NDMI 850 (light orange)

We assessed the predictive accuracy of NDMI 450 of DEX use in a subset of the samples used for the correlation analyses (Fig. 3). NDMI 450 score was highly predictive of DEX exposure in the IPS training set (AUROC: 0.99; 95% CI 0.99, 1), similar to the performance of the NDMI 850 score (AUROC: 0.99; 95% CI 0.99, 1). Using the same AGS test set (*n* = 552) used to evaluate NDMI 850, where it was highly predictive of DEX exposure (AUROC: 0.92; 95% CI 0.88, 0.96), the NDMI 450 score performed similarly (AUROC: 0.92; 95% CI 0.89, 0.96). For the AGS pilot cases, AUROCs from both NDMI 450 and NDMI 850 were 1 (95% CI 1–1). Using the likelihood ratio test for comparing nested logistic models of the NDMI 450 and NDMI 850 score predicting DEX exposure, there was no evidence that the fit of the smaller model (with only the NDMI 450 score) differed from that of the larger model for the given data. Thus, the models fit using both the NDMI 450 and NDMI 850 scores as covariates do not fit the data significantly better than models fit using only the NDMI 450 score as a covariate in the IPS training set (*χ*^2^ = 1.43, *p* = 0.23) and the AGS test set (*χ*^2^ = 0.35, *p* = 0.55).

**Fig. 4:**
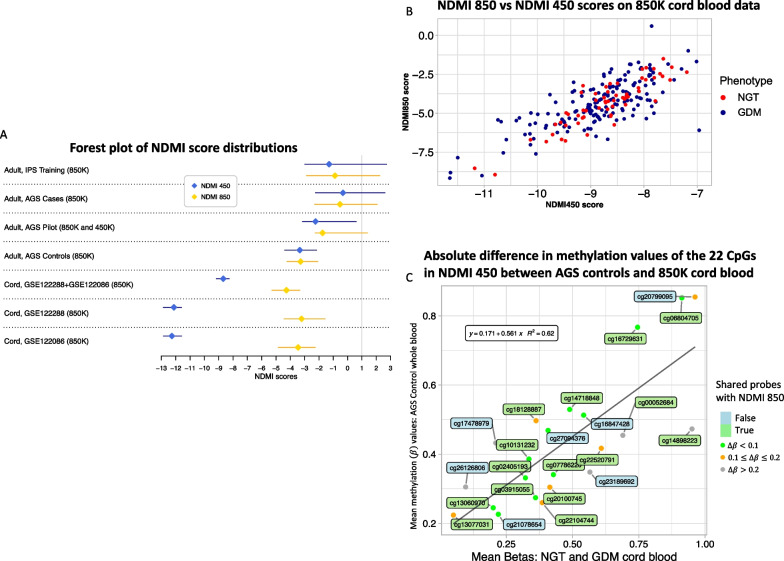
On the 850K array, NDMI 450 and NDMI 850 scores were consistent in whole blood. In cord blood on the 850K array, NDMI scores between the two algorithms were very different. NDMI 450 scores were a lot lower than that of AGS controls, but NDMI 850 scores in cord blood were relatively similar to that of AGS controls. **A** The first four rows denote the adult whole blood samples from the AGS and IPS studies that were run on the 850K array. The NDMI 450 and NDMI 850 score distributions are similar regardless of platform. In the next three rows, with the first one being the 850K cord blood datasets combined for the purpose of analyzing phenotypes of NGT and GDM, NDMI 450 and NDMI 850 score distributions are significantly different from each other, but NDMI 850 scores from cord blood are similar to that of the adult whole blood controls. The difference between the two score distributions is less extreme when the 2 850K datasets are combined. **B** Scatterplot of NDMI 450 and NDMI 850 in the combined 850K cord blood dataset with moderate correlation (*r* = 0.77, *p* < 0.0001), but NDMI 450 scores are more negative. Normal glucose tolerance (NGT) samples are denoted in red, and gestational diabetes mellitus (GDM) in blue. **C** The absolute differences in methylation values of the 22 CpGs in NDMI 450 between the AGS controls (whole blood) and 850K cord blood. The 15 shared CpG probes with NDMI 850 are shown in green, and the 7 unshared probes are in blue. The color of the points denotes the degree of absolute differences

**Fig. 5:**
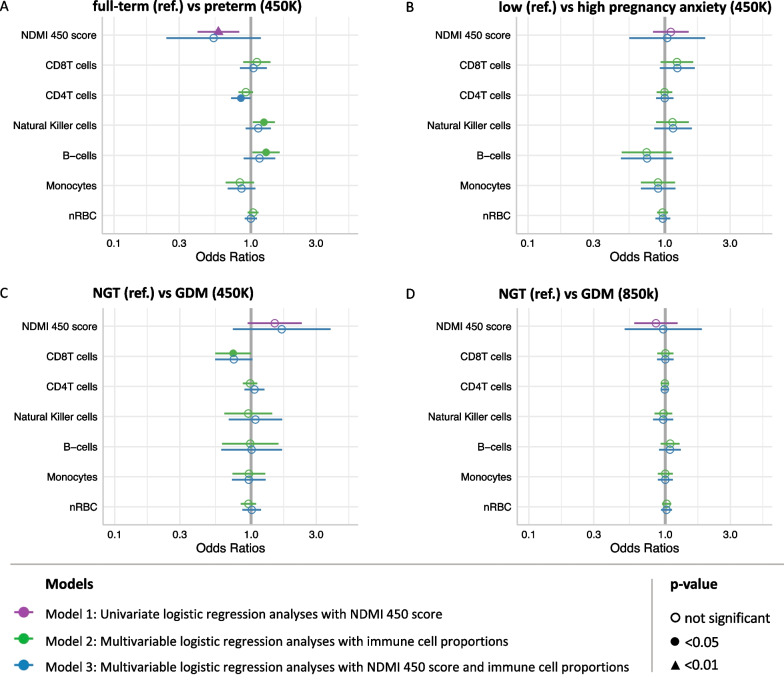
Comparison of NDMI 450 and immune profiles in predicting maternal risk factors from cord blood. Three logistic regressions were run on the GEO cord blood datasets, with the independent variable set as the phenotype, and NDMI 450 score (purple), immune cell proportions (green), or NDMI 450 and immune cell proportions (blue) as predictors. Significance is denoted by the shading and shape of the symbol for the model estimate. **A** GSE152380: 450K, full-term (ref.) versus preterm. NDMI 450 score was significant in the univariate model, and in the model with just cell proportions, NK cell and B cell proportions were significant in distinguishing full-term versus preterm. CD4T cell proportion was a significant predictor in the third model, but NDMI score was not. **B** GSE104376: 450K, newborns born to low (ref.) versus high anxiety individuals. **C** GSE153219: 450K, normal for glucose tolerance (ref.) versus gestational diabetes mellitus. In the second model, CD8T cell proportions were a significant predictor. **D** GSE122288, GSE122086: 850K, normal for glucose tolerance (ref.) versus gestational diabetes mellitus

*Identifying maternal risk factors in newborns using NDMI scores* The cord blood datasets consisted of comparing newborns born to individuals with low versus high anxiety, full-term versus preterm newborns, and newborns born to individuals with normal for glucose tolerance (NGT) versus GDM. In determining cutoffs for low and high anxiety, the upper and lower quartiles of the (pregnancy-related anxiety questionnaire) PRAQ-integrity scores were taken, and the mean (standard deviation) of the low and high anxiety individuals was 2.1 (0.4) and 5.2 (0.7), respectively [20]. NDMI 450 score distributions in the cord blood datasets available on the GEO database were significantly lower than that of whole blood samples from the AGS non-glioma adult controls (Table 1, Fig. 4A). Between these risk factors, NDMI 450 score distributions were not significantly different from the Wilcoxon rank-sum test except in GSE152380, the full-term versus preterm newborns dataset (*W* = 346, *p* = 0.002, Additional file 2: Supp. Table S3). The NDMI score distributions were significantly higher in full-term newborns than in preterm newborns (Additional file 1: Supp. Fig. S3). From logistic regression models with NDMI 450 score as a predictor, a one-unit increase in NDMI 450 score led to a 72% decrease in odds (OR: 0.58; 95% CI 0.39, 0.80, *p* = 0.002) of being a preterm newborn compared to a full-term newborn (Fig. 5, Additional file 2: Supp. Table S2). NDMI score was not a significant predictor in the other the cord blood datasets. For a one-unit increase in NDMI 450 score, there was an 11% increase (OR: 1.11; 95% CI 0.82, 1.52, *p* = 0.51) in the odds of having high pregnancy anxiety compared to the baseline of low pregnancy anxiety. There were conflicting results with the 450K dataset (GSE153219) and the combined 850K datasets (GSE122288, GSE122086) comparing NGT and GDM. There was a 49% increase (OR: 1.49; 95% CI 0.98, 2.47, *p* = 0.087) in the odds of having GDM in the 450K dataset with every unit increase in NDMI 450 score, whereas a 17% decrease (OR: 0.86; 95% CI 0.59, 1.22, *p* = 0.40) in the odds of being GDM compared to the baseline of NGT in the 850K dataset. Neither of these results was statistically significant.

**Table 1:** Characteristics and NDMI scores stratified by dexamethasone use (whole blood) or maternal risk factor (cord blood)

Study name/GEO series no	Immune profiles study (IPS)—pre-surgery glioma cases		Adult glioma study (AGS)—glioma cases		AGS—pilot cases		AGS—controls	GSE101840	GSE152380		GSE104376		GSE153219		GSE122288	GSE122086
Platform	850K		850K		850K, 450K		850K	450K	450K		450K		450K		850K	850K
Sample type	Whole blood		Whole blood		Whole blood		Whole blood	Cord blood	Cord blood		Cord blood		Cord blood		Cord blood	Cord blood
Disease state	Pre-surgery, pre-treatment glioma patients		195 IDH/1p19q/TERT-WT gliomas, 140 other gliomas		Pilot glioma cases		Adult non-glioma controls	Healthy newborn	Preterm birth		Pregnancy anxiety		Normal glucose tolerance (NGT) versus gestational diabetes mellitus (GDM)		Normal for glucose tolerance (NGT)	Gestational diabetes mellitus (GDM)
Phenotype of interest	No DEX at blood draw	DEX at blood draw	No DEX at blood draw	DEX at blood draw	No DEX at blood draw	DEX at blood draw	No DEX at blood draw	Cord blood controls	Full-term newborn	Preterm newborn	Low anxiety	High anxiety	NGT	GDM	NGT	GDM
N (missing phenotype)	79	59	184 (1)	150	6	6	447 (6)	6	72	18	22	23	26	16	61	165
Age [years]/gestational age [weeks] (mean (SD))	47.18 (12.64)	54.20 (17.18)	50.80 (15.79)	53.49 (14.33)	46.67 (6.77)	52.00 (2.76)	51.84 (15.48)	NA	34.77 (5.03)	33.27 (6.49)	39.61 (1.03)	39.59 (1.46)	NA	NA	38.74 (1.24)	38.76 (1.22)
Normal delivery	NA	NA	NA	NA	NA	NA	NA	0 (0%)	53 (74%)	18 (10%)	20 (90%)	15 (65%)	NA	NA	NA	NA
*Sex*																
Female	29	24	81	63	3	2	207	4	39	7 (39%)	11	10	11	9	27	42
(col %)	(38%)	(41%)	(44%)	(42%)	(50%)	(33%)	(46%)	(67%)	(54%)	(39%)	(50%)	(43%)	(42%)	(56%)	(44%)	(25.5%)
Male	47	35	103	87	3	4	246	2	33	11	11	13	15	7	34	123
(col %)	(62%)	(59%)	(56%)	(58%)	(50%)	(67%)	(54%)	(33%)	(46%)	(61%)	(50%)	(57%)	(58%)	(44%)	(56%)	(74.5%)
NDMI450 score (IQR)	− 2.78 (− 3.63, − 2.04)	3.27 (1.45, 4.61)	− 1.45 (− 3.10, 0.34)	2.24 (− 0.52, 4.33)	− 3.23 (− 3.44, − 2.86)	0.86 (− 0.33, 1.73)	− 3.34 (− 4.39, − 2.23)	− 5.79 (− 6.66, − 4.90)	− 5.87 (− 6.76, − 4.76)	− 7.52 (− 8.58, − 6.14)	− 5.97 (− 7.57, − 4.96)	− 5.18 (− 7.21, − 4.42)	− 6.07 (− 6.98, − 4.97)	− 5.43 (− 6.33, − 4.51)	− 8.67 (− 9.15, − 8.30)	− 8.71 (− 9.26, − 8.27)
NDMI850 score (IQR)	− 2.73 (− 3.60, − 1.94)	2.72 (1.48, 4.15)	− 1.45 (− 3.01, 0.19)	1.49 (− 0.82, 3.36)	− 2.31 (− 3.24, − 2.15)	1.66 (0.90, 3.18)	− 3.28 (− 4.23, − 2.08)	NA	NA	NA	NA	NA	NA	NA	− 4.18 (− 5.19, − 3.30)	− 4.3 (− 5.30, − 3.36)

*NA* not available

Granulocytes were detected with high variance inflation factors (VIF) and removed due to multicollinearity. Therefore, the final logistic models included the 6 immune cell proportions used in the logistic models that included only cell proportions and the NDMI score (Additional file 2: Supp. Table S2). When only cell proportions were included in the model, an increase in NK cells (OR: 1.25; 95% CI 1.05, 1.52, *p* = 0.018) and B cells (OR: 1.29; 95% CI 1.05–1.67, *p* = 0.027) was independently associated with higher odds of being preterm newborn, and the effects of NK and B cells were no longer observed. When NDMI 450 score was also included in the model, an increase in CD4T proportions was associated with a decrease in the odds of being a preterm newborn (OR: 0.85; 95% CI 0.71–0.99, *p* = 0.045). In the 450K dataset of NGT and GDM, an increase in the proportion of CD8T cells was associated with a decrease in the odds of having gestational diabetes mellitus (OR: 0.74, 95% CI 0.53–0.98, *p* = 0.047). In logistic models of immune cell profiles including or excluding the NDMI score, no cell subset proportions were found to be significant predictors of NGT and GDM in the 850K dataset.

*Cross-platform compatibility of NDMI in cord blood data* For the combined 850K datasets of gestational diabetes mellitus (GSE122086) and normal for glucose tolerance (GSE122288), NDMI 450 score distributions were significantly lower than the NDMI 850 score distributions (Kolmogorov–Smirnov *D* = 0.958, *p* < 0.0001, *D* = 0.967, *p* < 0.0001, respectively, Additional file 2: Supp. Table S3). There was a moderate correlation between NDMI 450 and NDMI 850 scores (*r* = 0.77, *p* < 0.0001, Fig. 4B) in the combined dataset. The NDMI 850 score distributions were slightly more positive than that of the other 450K GEO datasets. Assessment of mean methylation (*β*) between whole blood controls and the 850K cord blood showed that: of the 15 shared CpG probes, 8 showed beta differences < 0.1, 5 between 0.1 and 0.2, and 2 > 0.2 (Fig. 4C).

## Discussion

The goal of this study was to evaluate the NDMI biomarker on the 450K array using the same set of glioma patients and controls to train and evaluate the original biomarker built on the 850K array. In addition, we compared the two biomarkers in other clinical scenarios and applied the NDMI 450 to explore the glucocorticoid pathway in other datasets. We first evaluated the NDMI 850 with only the 15 overlapping probes on the 450K array, but the NDMI scores showed to be biased from using only the overlapping probes, confirming the need to rebuild NDMI on the 450K array. From the analyses performed, we observed high correlations between NDMI 450 scores and NDMI 850 scores, as well as cross-platform compatibility in both glioma patients and non-glioma controls. NDMI 450 was shown to be an accurate predictor of DEX use at blood draw in adult whole blood. The NDMI 450 should be considered in the clinical setting of dosing glucocorticoid treatment, as it requires fewer probes compared to NDMI 850 to identify variabilities in DEX response. Further, the NDMI 450 biomarker may be a valuable marker to include in analyses of biobank and archived methylation 450K data for samples collected from patients with other diseases and conditions where glucocorticoids are widely used, e.g., infectious diseases, non-glioma cancers, autoimmune conditions, and organ transplantation.

Newborn cord blood is inherently different from adult whole blood, so differences in NDMI scores comparing samples of each are not surprising. However, differences between NDMI 450 and NDMI 850 scores in the same dataset were not expected. To further explore the utility of NDMI 450 in determining reproductive outcomes, we analyzed data from cord blood to assess NDMI 450’s ability to identify maternal risk factors involved in the glucocorticoid pathway and factors that could lead to adrenal insufficiency in newborns. Our results showed that NDMI 450 was not associated with pregnancy anxiety (low vs. high, GSE104376) or with glucose tolerance during pregnancy (NGT vs. GDM, GSE153219, GSE122288/GSE122086). Results from the 450K and 850K datasets of GDM and NGT were inconsistent; NDMI 450 scores in the 450K dataset were higher in GDM than in NGT, whereas NDMI 450 scores in the 850K datasets were similar between the phenotypes. Though the NDMI 450 score was not statistically significant in either of these datasets, there was a trend toward significance in the 450K dataset. Finally, results suggest that NDMI 450 scores might be useful to distinguish full-term versus preterm newborns (GSE152380).

Preterm newborns are at higher risk of adrenal insufficiency due to the immaturity of the adrenal gland and reliance on the mother for cortisol until the third trimester, and they also have lower cortisol stress reactivity compared to full-term infants when subject to medical procedures at 4 months, which may explain the lower NDMI score distributions [21, 22]. Higher NDMI score distributions in full-term newborns could be attributed to fetal exposure to higher maternal cortisol during the third trimester, which is critical for neurodevelopment. There is insufficient evidence to support routine use of glucocorticoids for preterm and full-term newborns with low cortisol, especially given the heterogeneity in severity and potential adverse events. Glucocorticoid treatment in extremely preterm infants can result in spontaneous gastrointestinal perforation from hydrocortisone therapy, whereas some term infants require treatment that may be due to their transition to extrauterine life, and for other late preterm and full-term infants treatment is even more unclear [21]. As records of exogenous antenatal corticosteroids were not available, further evaluation of NDMI 450 in other publicly available cord blood datasets is warranted to help determine its potential clinical utility for predicting glucocorticoid-induced adrenal insufficiencies and serving as a tool to aid in glucocorticoid dosage decisions for mothers and infants.

The different maternal risk factors, including GDM, pregnancy anxiety, and preterm birth, may play a role in altering immune profiles. Our analyses of immune cell profiles showed that decreased odds of being a preterm infant were associated with increased CD4T cells when NDMI 450 was accounted for, and with increased NK and B cell proportions when NDMI 450 was not accounted for. This conflicts with published literature reports that preterm infants exhibited reduced frequencies of CD56^bright^ NK cells and increased frequencies of CD4+ T cells and transitional B cells [19]. Additionally, our analyses of glucose tolerance showed an increase in CD8T proportions was associated with a significant decrease in odds of GDM. However, CD8T frequencies were not associated with GDM in previous published studies [18].

Current publicly available data are significantly limited by a lack of clear measures of adrenal activity that are required to confirm a role of NDMI in glucocorticoid pathways in cord blood and other patient samples. What may be interesting to note is that when measuring the same loci in the 850K array, NDMI 850 scores of the adult non-glioma controls are similar to that of the cord blood, yet NDMI 450 is very different between the two samples. The difference cannot be attributed to platform type, as the 450K and 850K array produced similar NDMI scores and deconvoluted cell proportions. In addition, the 22 selected loci of NDMI 450 do not overlap with epigenetic clock CpGs or CpGs associated with the fetal cell origin signature, FCO, and differences in methylation between whole blood and cord blood were not specific to either the shared or non-shared probes with NDMI 850 [23–25]. Cross-platform compatibility was not observed in cord blood samples with the NDMI biomarker.

Although potential clinical implications have been established with NDMI for glioma, its application in other contexts will need further studies including clinical trials, to assess the clinical value of NDMI in cord blood and other sample types. Limitations associated with pooling publicly available data, including sequencing from different laboratories and missing covariates, precluded our ability to adjust for batch effects in the GEO datasets without losing the ability to detect biological differences in our analyses. Clinical trials studying maternal stress or antenatal glucocorticoid treatment in addition to short- and long-term effects on development are needed to better assess NDMI in a cord blood setting although ethical challenges currently prevent such studies.

## Conclusions

We have developed an algorithm that reproduces the glucocorticoid response score using 450K data for glioma patients and controls. This may increase the accessibility for researchers to assess glucocorticoid response in archived or publicly available datasets that use the 450K version of the Illumina BeadChip array. However, since the NDMI 450 and NDMI 850 were not concordant in samples from cord blood, applications of NDMI to other samples and array data require further clarification.

## Methods

### Patient and control samples

The UCSF Immune Profiles Study (IPS) is a prospective longitudinal study of glioma patients pre- and post-surgery, with the goal of collecting clinical, blood, and MRI data over multiple time points, including, before treatment/pre-surgery, before treatment/after surgery, after treatment/after surgery, and at each subsequent follow-up MRI. Treatment refers to the GBM standard of care (surgery, chemotherapy, and radiation). All patients who have their blood samples drawn are given a questionnaire upon consent to participate, which asks for information on comorbidities, demographics, and medication use. Blood samples are obtained either at UCSF (at the phlebotomy clinics, by the hospital phlebotomist or UCSF Clinical Research Center, or at the UCSF radiology clinics) or at outside blood draw laboratories (through mailed blood kits). Data and sample collection are ongoing at UCSF; blood is collected at each follow-up blood collection, which usually coincides with scheduled MRIs or doctor’s appointments. Patients are followed until drop out, loss to follow-up, tumor progression, death, or other competing events. The training dataset derived from this study consists of 135 pre-surgery glioma patients, of which 59 were exposed to DEX, and 76 were not.

The test set is based on patients in the Adult Glioma Study (AGS), a case–control study of patients recruited from 1991 to 2013 [26–30]. The study consisted of five recruitment series, including population- and clinic-based cases and controls. Questionnaires were administered by study interviewers either in-person or by phone. A blood sample was collected at the time of interview by a study phlebotomist, usually a few months after the glioma surgery. Only one blood sample was obtained per patient, usually after surgery, compared to multiple blood draws for IPS. From the AGS participants, the test dataset was formed from with 195 IDH/1p19q/TERT-WT gliomas, 140 other gliomas, and 453 non-glioma controls. 150 of the AGS glioma samples were exposed to DEX, and 184 glioma samples were not, with 1 missing DEX status. In the 453 AGS controls, 6 were missing DEX status. The IPS and AGS data are all run on the 850K array. There are also pilot samples available from the AGS study, 12 of which were run on the 450K and 850K array. These 12 samples were also included in the analysis.

### Public data

This study uses Illumina HumanMethylation450 and HumanMethylationEPIC BeadChip methylation data available at the Gene Expression Omnibus (GEO) Database under accession GSE104376, GSE152380, GSE153219, GSE101840, GSE122086, and GSE122288. GSE104376, GSE152380, GSE153219, and GSE101840 were profiled on the Infinium Methylation450 platform (GPL13534), and GSE122086 and GSE122288 were profiled on the Infinium MethylationEPIC platform (GPL21145). These were all cord blood samples, with 183 samples on the 450 platform (450K), and 226 samples on the EPIC platform (850K).

### Processing DNA methylation array

Blood samples obtained from IPS and AGS were frozen at − 80 °C until DNA isolation and bisulfite conversion for the Illumina MethylationEPIC BeadChip arrays. The 12 AGS pilot samples were also run on the Illumina Methylation450 BeadChip array [31]. In both adult whole blood and cord blood samples, preprocessing remained the same. The *minfi* Bioconductor package for preprocessing and quality control of the methylation data was obtained from ratios of the fluorescent signals in methylated versus unmethylated loci [32]. If the detection p values for a particular CpG locus were above the predetermined threshold (*P* > 10E−5) in more than 25% of the samples, that locus was consequentially removed. A ‘noob’ background correction (normal-exponential convolution using out-of-band probes) and dye bias equalization were applied to correct for background fluorescence and dye bias within the array [33]. Beta-mixture quantile normalization (BMIQ) was then applied to correct for probe design bias. CpG sites on sex chromosomes, single nucleotide polymorphisms (SNPs), and probes targeting non-CpG sites (ch probes) were removed from the data. For GEO datasets with missing data on sex, the predicted sex was obtained from the methylation data using the *getSex* function in the *minfi* package. The two GEO datasets of gestational diabetes mellitus (GSE122086) and normal for glucose tolerance (GSE122288) that were both run on the 850K platform by the Department of Maternal–Fetal Biology at the National Research Institute for Child Health and Development in Japan were processed separately, but merged together for NDMI score and phenotype analysis. No ComBat or imputation was performed.

### Reference-based deconvolution of whole blood and cord blood samples

The optimized reference-based library was used to deconvolute the 6 immune cell proportions (CD4^+^ T cells, CD8^+^ T cells, B cells, natural killer cells (NK), monocytes (mono), neutrophils (neu)) from whole blood and 7 immune cell proportions in cord blood (CD8^+^ T, CD4^+^ T, NK, B cells, monocytes, granulocytes (gran), nucleated red blood cells (nRBC)) from cord blood [34]. 

### Reconstructing the neutrophil dexamethasone methylation index (NDMI) on the Illumina HumanMethylation450 BeadChip

The neutrophil dexamethasone methylation index (NDMI), a 28 neutrophil-specific loci classifier of dexamethasone (DEX) exposure, was built using 2621 CpGs that showed a neutrophil-specific association with DEX exposure that is available on the Illumina HumanMethylationEPIC BeadChip array. These probes were identified by applying CellDMC, a statistical algorithm that conducts cell-specific DNA methylation analyses based on bulk tissue DNA methylation profiles, in glioma patients who had not received any surgery, chemotherapy, or radiation in the Immune Profiles Study (IPS) [14, 35]. Fifty-nine of these patients were DEX exposed, and 76 were non-exposed, with exposure described as DEX use at the time of blood draw (*N* = 52) or DEX use within the past 30 days (*N* = 7). 897 of these 2621 neutrophil-specific loci were available on the 450K array. To reconstruct this NDMI biomarker, the same set of IPS patients were run on elastic net regression, fit with the 897 probes using the *glmnet* package in R. The model selected a subset of loci to construct the NDMI classifier. Associated target gene names to the chosen predictors were identified using the *getAnnotation* function from the *IlluminaHumanMethylation450kanno.ilmn12.hg19* package and the UCSC Genome Browser that references the Genome Build 37 (GRCh37/hg19). The resulting elastic net model was tested on a subset of the AGS dataset, which consisted of 195 IDH/1p19q/TERT-WT gliomas, 140 other gliomas, and 453 non-glioma controls. The linear composite score calculated from the coefficients of the selected neutrophil-specific loci from the elastic net, termed the NDMI score, was calculated for the reconstructed NDMI on the 450K array. We refer to the 450K NDMI algorithm and its linear composite score as NDMI 450 and NDMI 450 score, respectively, and for the original NDMI algorithm and its score as NDMI 850 and NDMI 850 score, respectively. We also reduced the NDMI 850 to the 15 CpGs that were available on the 450K array (reduced NDMI 850) and compared its score distributions to that of NDMI 850. To compare the NDMI scores and their respective predictive performance, we performed Bland–Altman analysis, calculated Pearson correlations on the same set of subjects, and plotted receiver operating characteristic (ROC) curves to compare NDMI 450’s classification accuracy of DEX use in the training and test datasets with that of NDMI 850.

### Assessing cross-platform compatibility with receiver operating characteristic curve (ROC) and correlation analyses

To assess the correlation and agreement of the NDMI 450 to the ‘gold standard,’ the NDMI 850 biomarker, we estimated Pearson correlation coefficients among the IPS pre-surgery glioma samples, the AGS pilot cases, combined AGS glioma cases, and the AGS controls. We also conducted Bland–Altman analysis of the NDMI scores in the same samples. ROC curves were plotted to analyze the classification accuracy of dexamethasone use at blood draw. In addition to the IPS and AGS samples in the correlation analysis, we used the same 552 AGS cases and controls to compare the predictive accuracy of DEX use at blood draw in NDMI 450. The likelihood ratio test was performed to assess differences in the predictive accuracy of the NDMI 450 score and NDMI 850 score.

### Evaluating NDMI 450 in cord blood data

Anxiety was determined through a pregnancy-related anxiety questionnaire (PRAQ) regarding the parent’s assessment of newborn health that provided scores from zero to seven, and the low and high anxiety individuals were selected based on the lowest and highest quartiles, respectively [20]. For GEO cord blood datasets with two phenotypes available, NDMI 450 score distributions were compared for significant differences using the Wilcoxon rank-sum test. NDMI 450 score distributions from all GEO cord blood datasets were compared to that of the adult AGS control samples using Wilcoxon rank-sum tests. For the 2 cord blood datasets run on the 850K array, NDMI 850 scores were also compared. To evaluate whether NDMI score is associated with the phenotype of each cord blood dataset, logistic regression models were implemented with NDMI scores as the predictors and phenotype as the outcome. Additional logistic regression models including deconvoluted cell proportions with and without NDMI score were also performed. In the two 850K cord blood datasets, cross-platform compatibility was determined with the Kolmogorov–Smirnov test, correlation analysis, and comparison of logistic regressions between the NDMI 450 and NDMI 850 scores. The absolute differences in mean methylation values of the 450K probes selected by the elastic net regression were calculated between the AGS adult controls and the 850K cord blood samples.

## Supplementary Information

**Additional file 1: Figures.** Supplemental figures of reduced NDMI 850 in pilot samples, with flow of array and sample comparisons, Bland–Altman analysis, and distributions of NDMI scores by phenotype in the GEO cord blood datasets.

**Additional file 2: Tables.** CpGs in NDMI 450 and whether they were shared with NDMI 850. Model coefficients from logistic models on risk factors in pregnancy; Table of model coefficients of the three logistic models run on GEO cord blood data. With maternal risk factor set as the outcome variable for all three models, the predictor of the first model was NDMI 450 score, the second immune cell proportions, and the third immune cell proportions and NDMI 450 score.

## Data Availability

Methylation and phenotype data used in this manuscript will be available through dbGaP Study Accession phs001497.v2.p1 for the Adult Glioma Study. Methylation and phenotype data from the Immune Profiles Study will be available through dbGaP Study Accession phs002998.v1.p1. Requests to access additional deidentified data used in this manuscript will be considered through a request to the corresponding author. The datasets supporting the conclusions of this article are available on the Gene Expression Omnibus (GEO) Database under accession GSE104376, GSE152380, GSE153219, GSE101840, GSE122086, and GSE122288.

## Abbreviations

AGS: Adult Glioma Study
BA: Bland–Altman
DEX: Dexamethasone
GC: Glucocorticoid
GR: Glucocorticoid receptor
GBM: Glioblastoma
CpG: Cytosine phosphate guanine dinucleotide
450K: Infinium Methylation450 BeadChip
850K: Infinium MethylationEPIC BeadChip
LoA: Limits of agreement
NDMI: Neutrophil dexamethasone methylation index
OR: Odds ratio
IDH: Isocitrate dehydrogenase
WHO: World Health Organization
WT: Wild type/non-mutant
