# Supplementary material for: Evaluation of cross-platform compatibility of a DNA methylation-based glucocorticoid response biomarker
Source: Clin Epigenetics. 2022 Oct 28;14:136. doi: 10.1186/s13148-022-01352-1 (PMC9617416; doi:10.1186/s13148-022-01352-1)

Supplemental Figure 1. Reduced NDMI 850 scores were biased in comparison to NDMI 850 scores. (A) Workflow of obtaining NDMI 450, NDMI 850, and the reduced NDMI 850 with just the 15 overlapping probes on the 450K array. (B) Scatter of the NDMI 850 scores compared to the reduced NDMI 850 scores. While correlations were high (r=0.99, p<0.0001), reduced NDMI 850 scores were more positive than that of NDMI 850 scores. (C) Workflow of the different NDMIs we observed and how they were compared across the datasets used.

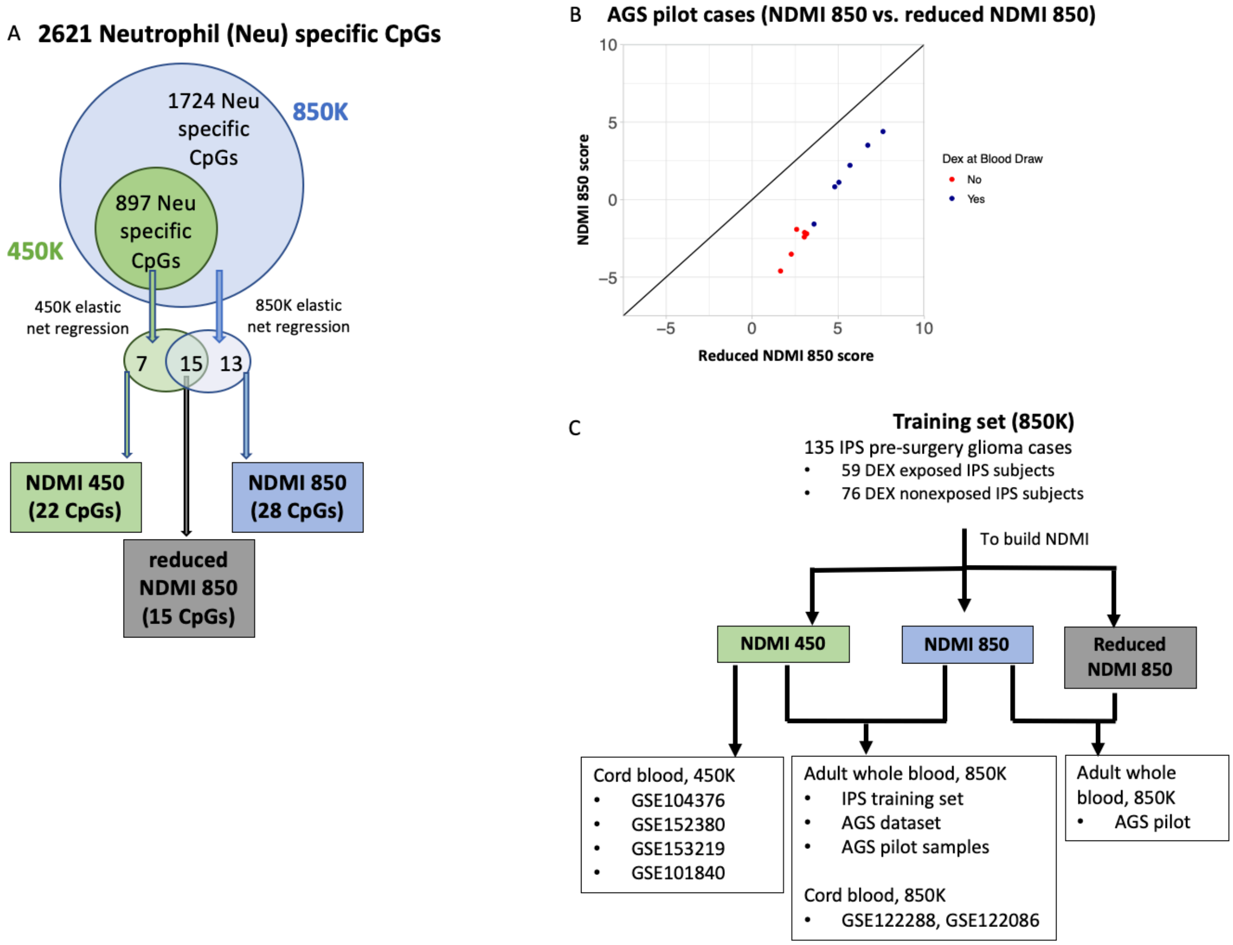

Supplemental Figure 2. Bland-Altman analysis of NDMI 450 and NDMI 850 scores in the IPS and AGS samples. Log and squared transformations to the NDMI scores were considered for the data, though untransformed data provided to be the best for this particular analysis. We observed that as the average in NDMI scores increased, the NDMI 450 scores tended to overestimate relative to the NDMI 850 scores. This increasing trend was seen in both IPS pre-surgery samples and the AGS glioma cases, with higher NDMI scores in those taking DEX at blood draw. NDMI 850 was set as the gold standard, with the average of the two scores set as the x-axis. NDMI 450 scores tended to overestimate NDMI 850 scores as the average increased. Those who have not taken DEX at blood draw are in red, and those who have taken DEX at blood draw are in blue. (A) IPS pre-surgery cases - Bias: 0.089 (95% CI: -0.0048, 0.18); LLoA: -0.99 (95% CI: -1.15, -0.83); ULoA: 1.17 (95% CI: 1.01, 1.33); (B) AGS glioma cases - Bias: 0.37 (95% CI: 0.30, 0.45); LLoA: -0.97 (95% CI: -1.09, -0.84); ULoA: 1.72 (95% CI: 1.59, 1.84); (C) AGS pilot cases – Bias: -0.62 (95% CI: -1.1, -0.11); LLoA: -2.2 (95% CI: -3.1, -1.3); ULoA: 0.94 (95% CI: 0.051, 1.8); (D) AGS controls - Bias: -0.20 (95% CI: -0.061, 0.020); LLoA: -0.88 (95% CI: -0.95, -0.81); ULoA: 0.84 (95% CI: 0.77, 0.91).

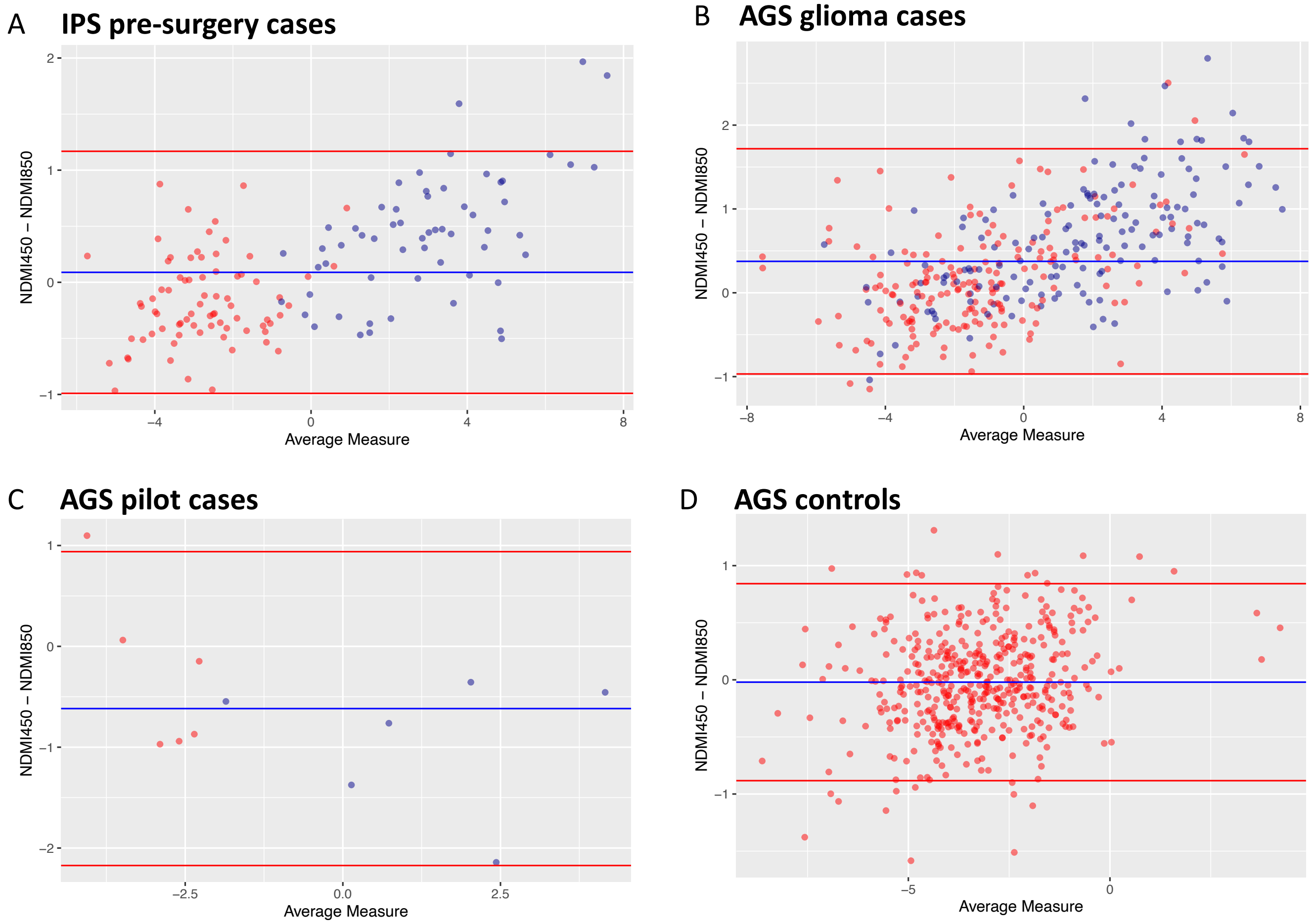

Supplemental Figure 3. Distributions of NDMI scores by type of maternal risk factors. (A) GSE101840: 450K, NDMI 450 scores in healthy cord blood samples; (B) GSE104376: 450K, NDMI 450 scores in low (ref.) vs high pregnancy anxiety; (C) GSE152380: 450K, NDMI 450 scores in full-term (ref.) vs preterm newborns; (D) GSE153219: 450K, NDMI 450 scores in normal for glucose tolerance (ref.) vs gestational diabetes mellitus. (E) GSE122288, GSE122086: 850K, NDMI 450 scores in normal for glucose tolerance (ref.) vs gestational diabetes mellitus; (F) GSE122288, GSE122086: 850K, NDMI 850 scores in normal for glucose tolerance (ref.) vs gestational diabetes mellitus.

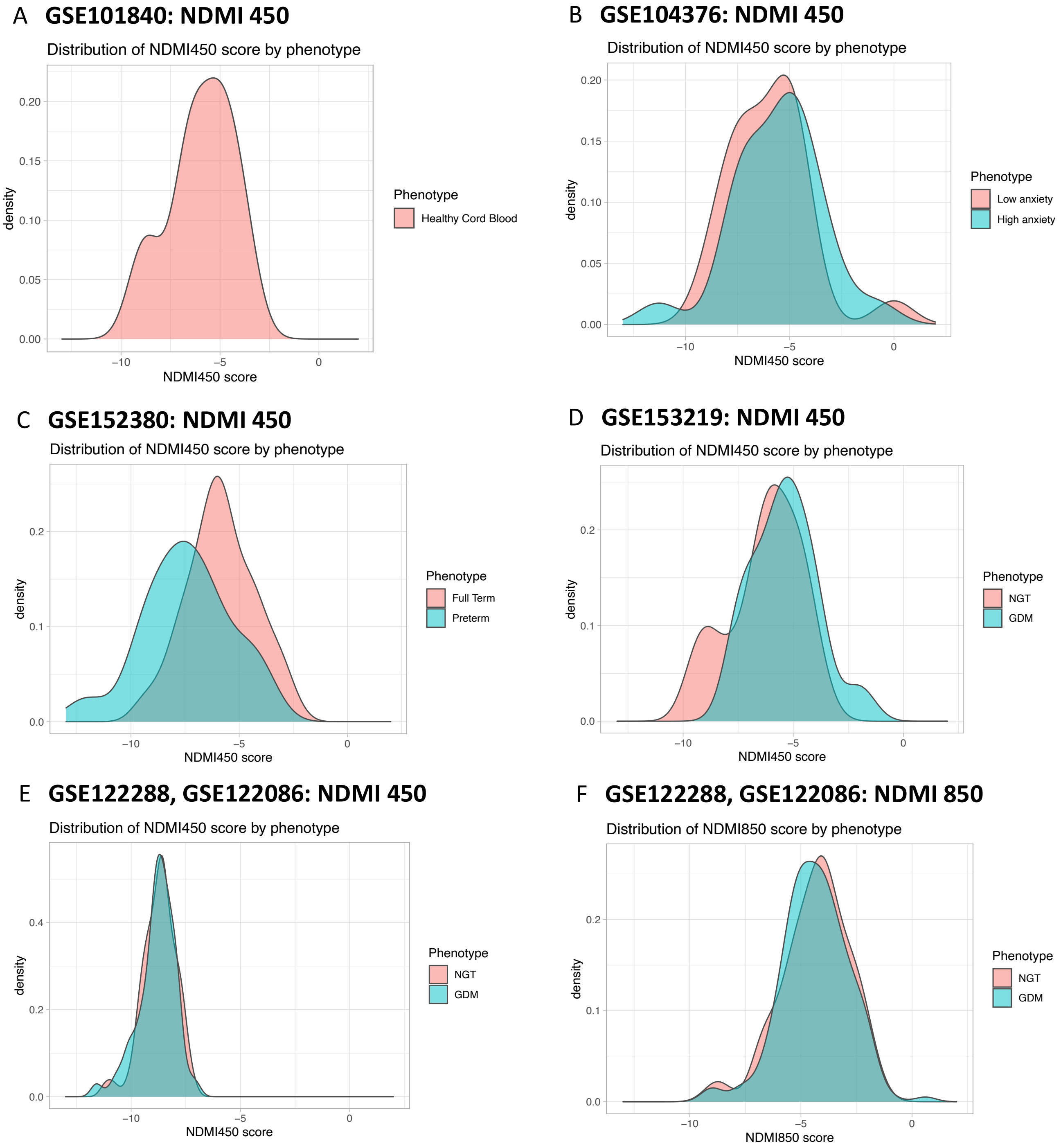

Supplement: Supplementary file 1 — Additional file 1: Figures. Supplemental figures of reduced NDMI 850 in pilot samples, with flow of array and sample comparisons, Bland–Altman analysis, and distributions of NDMI scores by phenotype in the GEO cord blood datasets. [file 13148_2022_1352_MOESM1_ESM.pdf]
